# Sympathetic Denervation-Induced MSC Mobilization in Distraction Osteogenesis Associates with Inhibition of MSC Migration and Osteogenesis by Norepinephrine/adrb3

**DOI:** 10.1371/journal.pone.0105976

**Published:** 2014-08-21

**Authors:** Zhaojie Du, Lei Wang, Yinghua Zhao, Jian Cao, Tao Wang, Peng Liu, Yabo Zhang, Xinjie Yang, Xiaobing Cheng, Baolin Liu, Delin Lei

**Affiliations:** 1 State Key Laboratory of Military Stomatology, Department of Oral and Maxillofacial Surgery, School of Stomatology, the Fourth Military Medical University, Xi’an, China; 2 Department of Oral and Maxillofacial Surgery, No. 425 Hospital of PLA, Sanya, China; 3 Department of Prosthodontics, Stomatology Hospital of Xi’an Jiaotong University, Xi’an, China; Université Jean Monnet, France

## Abstract

The sympathetic nervous system regulates bone formation and resorption under physiological conditions. However, it is still unclear how the sympathetic nerves affect stem cell migration and differentiation in bone regeneration. Distraction osteogenesis is an ideal model of bone regeneration due to its special nature as a self-engineering tissue. In this study, a rat model of mandibular distraction osteogenesis with transection of cervical sympathetic trunk was used to demonstrate that sympathetic denervation can deplete norepinephrine (NE) in distraction-induced bone callus, down-regulate β3-adrenergic receptor (adrb3) in bone marrow mesenchymal stem cells (MSCs), and promote MSC migration from perivascular regions to bone-forming units. An *in* *vitro* Transwell assay was here used to demonstrate that NE can inhibit stroma-derived factor-1 (SDF-1)-induced MSC migration and expression of the migration-related gene *matrix metalloproteinase-2* (*MMP-2*) and downregulate that of the anti-migration gene *tissue inhibitor of metalloproteinase-3 (TIMP-3)*. Knockdown of adrb3 using siRNA abolishes inhibition of MSC migration. An *in vitro* osteogenic assay was used to show that NE can inhibit the formation of MSC bone nodules and expression of the osteogenic marker genes *alkaline phosphatase (ALP)*, *osteocalcin (OCN)*, and *runt-related transcription factor-2 (RUNX2)*, but knockdown of adrb3 by siRNA can abolish such inhibition of the osteogenic differentiation of MSCs. It is here concluded that sympathetic denervation-induced MSC mobilization in rat mandibular distraction osteogenesis is associated with inhibition of MSC migration and osteogenic differentiation by NE/adrb3 *in vitro*. These findings may facilitate understanding of the relationship of MSC mobilization and sympathetic nervous system across a wide spectrum of tissue regeneration processes.

## Introduction

Current bone regeneration methods are still unable to satisfactorily correct large bone deformities and defects. This is largely because of poor understanding of stem cell mechanisms. Distraction osteogenesis (DO) has been widely used to regenerate bone tissues for deformities and defects in long bones and maxillofacial bones. DO induces the formation of new bone along gradual distraction stress at the broken ends of bone. It is an ideal model of bone regeneration because of its special nature as a self-engineering tissue. Understanding the stem cell mechanism may help shorten the treatment period of DO, which is somewhat long, thereby minimizing inconvenience to patients and reducing the rate of clinical complications [1]–[4]. Osteoblasts are derived from mesenchymal stem cells (MSCs) in adjacent bone marrow and periosteum under the mechanical tension in DO, and massive osteoblast recruitment can lead to bone regeneration [5]–[8]. It has been demonstrated that MSCs are a subset of perivascular cells and reside in particular microenvironments called niches. These control MSC survival, proliferation, self-renewal, and differentiation [9]–[15]. In order to differentiate into osteoblasts, MSCs must migrate from their niches to bone-forming sites. For this reason, MSC mobilization is prerequisite to the bone formation in DO.

The sympathetic nervous system has been shown to innervate the skeletal system and to regulate bone formation and resorption, thereby maintaining bone homeostasis [16]–[18]. It has been shown that norepinephrine (NE), a major mediator of the sympathetic nervous system, influences osteoblasts both in culture and *in vivo* [19]–[22]. NE has also been demonstrated to stimulate osteoclast formation, thereby enhancing bone resorption [19]. However, it remains unclear whether NE regulates MSC mobilization in bone regeneration processes, such as DO. Sympathetic fibers work with intraosseous vessels to regulate the mobilization of hematopoietic progenitor cells, and sympathetic denervation causes pericyte loss in retina [23]–[25]. It is therefore entirely possible that sympathetic nerves form part of the perivascular stem cell niche that MSCs occupy in the bone marrow.

One study by the present team recently showed that sympathetic denervation improves the quality of bone in rat mandibular DO [26]. Because MSCs express β_3_-adrenergic receptor (adrb3), it is here hypothesized that sympathetic nerves secrete neurotransmitter NE to confine MSCs to their niches *via* adrb3 [27]. The present study is the first to show, that the sympathetic nervous system may regulate MSC mobilization *via* norepinephrine/adrb3 *in vitro.* This study also shows that sympathetic denervation causes deleption of NE and promotion of MSC migration from perivascular sites to bone-forming units in DO. Additionally, NE may directly inhibit osteogenic differentiation of MSCs *via* adrb3 *in vitro*.

## Materials and Methods

### Animals and groups

**Figure 1:**
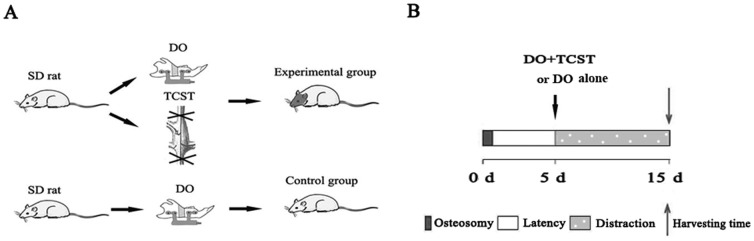
Groupings for rat mandibular distraction osteogenesis (DO). (**A**) Sprague-Dawley rats in the DO alone group received DO treatment, and those in the DO+transection of cervical sympathetic trunk (TCST) group underwent both DO and TCST treatment (n = 5, both). (**B**) After a 5-d latency, the distraction was initiated and allowed to take place for 10 days. Samples were harvested on day 10.

The animal experiment protocol was approved by the Committee on Use of Live Animals for Teaching and Research of the Fourth Military Medical University. Forty adult male Sprague-Dawley rats (280–320 g) were housed in a central animal research facility at a carefully maintained controlled temperature, controlled relative humidity, and a 12 h light/dark cycle in accordance with the guidelines established by the Animal Center of Fourth Military Medical University. Rats were randomly divided into 2 groups, an experimental group in which rats underwent DO+transection of cervical sympathetic trunk (TCST) and were killed after 10 d of distraction (n = 5) and a control group in which rats underwent DO alone and were killed after 10 d of distraction 10 d (n = 5) (Figure 1A, B). These experiments were repeated 3 times. Another 10 rats underwent no operation and were killed for immunofluorescence staining and MSC culture (n = 5 respectively).

### Surgical protocols

All experimental rats were injected intraperitoneally with 1% pentobarbital sodium (30 mg/kg) for anesthesia. Buprenorphine was administered subcutaneously (every 12 h for 48 h, 0.05 mg/kg) for post-operative analgesia.

DO procedure: After the rats were anesthetized, a 1 cm longitudinal submandibular incision was made along the right mandible body under aseptic conditions. The masseter muscle was dissected from the buccal ramus of the mandible. An osteotomy was created using a diamond disc from the upper border of the anterior ramus to the inferior border of the mandible and an external custom-made distraction device (Zhongbang Titanium Biomaterials Corporation, Xi’an, China) was inserted and fixed by two titanium screws on each side. Surgeries were performed carefully to prevent any injury to the inferior alveolar nerves. The incision was sutured, and the distraction rod was left exposed on the outside.

TCST procedure: The rats were anesthetized and a 1.5 cm longitudinal incision was made in the middle of the neck. The cervical muscles were separated and the bilateral carotid artery bifurcation was exposed at the level of the hyoid bone. The bilateral superior cervical ganglion was anatomized and resected by microsurgery in the back of the carotid arterial bifurcation.

After a latency period of 5 days, the rods were distracted gradually at 0.2 mm/12 h for 10 d. Rats in each group were killed by inhalation of carbon dioxide followed by decapitation. The mandible samples were harvested for laboratory analysis and all the measurements were conducted blindly.

### Bone mineral density analysis, immunofluorescence, and immunohistochemistry

The right mandibles and the distraction zone specimens were harvested and fixed in 4% paraformaldehyde in phosphate-buffered saline (PBS) at 4°C for 2 days. Bone mineral density analysis was performed as described previously [26]. Briefly, the samples were scanned with a high-resolution micro-computed tomography system (Inveon CT; Siemens AG, Munich, Germany) with a resolution of 1888×2048 pixels and an isotropic voxel size of 15 Km. The system was set to 80 kV, 500 mA, 800 ms integration time. After scanning, the micro-CT images were segmented using individual optimal thresholds to isolate bone tissue and obtain accurate three-dimensional data sets. The cuboid volume of interest in diameter of 2.2×1.5×1.5 mm was selected in the distracted gap for the bone mineral density assessment. For the purpose of mineral density calibration, a series of mineral reference phantoms made of fine calcium hydroxyapatite powder uniformly embedded in epoxy resin obtained from the micro-CT manufacturer were scanned prior to specimen scanning. CT values were converted into mineral density values using a linear calibration curve based on the gray values obtained from the mineral reference phantoms.

The specimens were decalcified in 20% ethylenediaminetetraacetic acid (EDTA) at 4°C for 2 to 4 weeks and embedded in paraffin. Longitudinal 5 µm serial sections were sliced, deparaffinized and subjected to immunofluorescence and immunohistochemisty. For immunofluorescence, the sections were incubated with anti-tyroxine-hydroxylase (TH, 1∶200, Santa Cruz Biotechnology, Santa Cruz, CA, U.S.), anti-nestin (1∶100, Santa Cruz) and anti-CD90 (1∶100, Sigma-Aldrich, St. Louis, MO, U.S.) primary antibodies overnight at 4°C. Goat anti-mouse IgG FITC and goat anti-rabbit TRITC (Santa Cruz) served as secondary antibodies (1∶200). Negative controls were isotypes with no known specificity tagged with the same fluorochromes as the test antibodies. For nuclear staining, 4′, 6-diamidino-2-phenylindole (DAPI, Sigma-Aldrich) was added to the slides after secondary antibody incubation, and incubated for 10 minutes at room temperature.

For immunohistochemisty, endogenous peroxidase activity was eliminated by using 3% hydrogen peroxide for 10 min at room temperature. The tissue slices were then permeabilized with 0.25% Triton X-100 in PBS for 30 min, and blocked in blocking solution (2% glycine, 2% bovine serum albumin, 5% fetal bovine serum, 50 mM NH 4Cl in PBS), for 1 h, at room temperature. The slices were then incubated with anti-NE (1∶500, Abcam, Cambridge, MA, U.S.), anti-adrb3 (1∶200, Santa Cruz), anti-stroma-derived factor-1 (SDF-1, 1∶200, Santa Cruz) or anti-nestin primary antibodies (1∶200, Santa Cruz) overnight at 4°C, followed by incubation with HRP-conjugated second antibodies (Santa Cruz) for 1 h at room temperature. The slides were washed and then developed using diamino-benzidine (DAB) as the chromogen. Brown yellow staining of the cytoplasmic membrane was considered indicative of binding. To quantify the ratios of nestin^+^ cells in bone forming areas per total number of nestin^+^ cells, bone forming areas consisting of fibrosis and woven bones were characterized by bone trabeculae and osteoid rimmed by osteoblasts within a bridging callus. Nestin^+^ cells were manually counted in 5 randomly selected high-power fields (400×) per slide under a microscope. Each experiment was performed in triplicate. Integrated optical density (IOD) of NE and adrb3 immunostaining of each group (n = 5) were measured using Image-ProPlus analysis software (Media Cybernetics, Inc., Rockville, MD, U.S.) in a blinded manner.

### Cell culture

The rat mandibles were harvested, and the attached soft tissues were removed and cut into small pieces. The cells were obtained by digestion with 3 mg/ml collagenase type I (Santa Cruz) and 4 mg/mL dispase II (Santa Cruz) for 60 min at 37°C. Single-cell suspensions were obtained through 70 µm cell strainers (BD Bioscience) and centrifuged at 500 g for 4 min. The cells were re-suspended with DMEM-low glucose (Hyclone Laboratories Inc., Logan, UT, U.S.), 10% FBS (Hyclone), antibiotics (100 units/mL penicillin G and 100 g/mL streptomycin) (HyClone), and seeded at 1×10^6^ on a 100 mm dish (Corning Inc., Corning, NY, U.S.), respectively. After 3 days of seeding, floating cells were removed, and the medium was replaced with fresh medium. Passages were performed when cells approached confluence. This was here considered passage 0 (P0). P0 and P1 cells were obtained for subsequent experiments.

### In vitro osteogenic assay

The P1 cells (2×10^5^) were seeded on 35 mm dishes (Corning) and cultured until they reached confluence. For osteogenic induction, MSCs were cultured in DMEM-low glucose containing 20% FBS, 2 mM β-glycerophosphate (Sigma-Aldrich), 100 µM L-ascorbic acid 2-phosphate (BD Bioscience, San Diego, CA, U.S.), 10 nM dexamethasone (Sigma-Aldrich), 2 mM L-glutamine, 55 µM 2-ME, and 100 U/mL penicillin/100 µg/mL streptomycin, in the absence or constant presence of 10^−7 ^mol/L NE (Sigma-Aldrich) for 4 weeks. A concentration of 10^−7 ^mol/L was selected because it was found to best approximate the normal concentration in *vivo*. MSCs were fixed with 4% PFA for 10 min and stained with 1% Alizarin red-S (Sigma-Aldrich) in distilled water for mineralized nodule detection. Eight representative fields at 40× magnification were selected per sample, and Alizarin Red-positive areas were analyzed using the NIH image software package Image-J and shown as the amount of Alizarin Red-positive area over total area. For knockdown of abrd3 expression in MSCs, siRNA transfection was performed according to the manufacturer’s instructions. Fluorescein-conjugated control siRNA served as a control and as a method of evaluating transfection efficacy. All siRNA products were purchased from Santa Cruz Biotech. Briefly, 2×10^5^ MSCs were plated into six well-plates and cultured for 24 h in antibiotic-free normal growth medium supplemented with 10% FBS. The cells were subjected to 4 h of serum starvation and then transfected with abrd3 siRNA or control siRNA for 12 h. The transfection mixture (media and siRNA) was then replaced with 20% FBS in normal growth medium and the sample was allowed to incubate for 72 h. The cells were further cultured for osteogenic analysis. The osteogenic assay was repeated with 5 independent isolated cells per experimental group.

### Western blot analysis

Total protein was extracted using M-PER mammalian protein extraction reagent (Thermo Fisher Scientific, Hudson, NH, U.S.). Protein was separated on 4–12% NuPAGE gel (Life Technologies, Grand Island, NY, U.S.) and transferred to ImmobilonTM-P membranes (EMD Millipore, Billerica, MA, U.S.). After blocking with 5% non-fat dry milk for 1 h, the membranes were incubated with adrb3 or nestin primary antibodies (1∶200 dilution) at 4°C overnight. Horseradish peroxidase-conjugated IgG (Santa Cruz) was used to treat the membranes for 1 h. They were then treated with a chemiluminescent substrate (Thermo). The bands were detected on Kodak X-ray film. Each membrane was stripped using a stripping buffer (Thermo) and reprobed with anti-β-actin to determine the loading amount.

### Chemotaxis assay

MSCs were incubated in the presence or absence of NE (10^−7 ^mol/L) and assayed for migration using a 24-well Transwell with a porous polycarbonate membrane insert (pore diameter 8 µm; BD Biosciences). Then 1×10^5^ cells in were placed in 200 µL serum-free medium. Each group of cells had either 10^−7 ^mol/L NE or no NE. These control cells were added to the upper chamber assembly. The lower chamber contained a concentration of 25 ng/mL SDF-1 in 600 µL of media. After 12 h of migration, the assay was terminated and the upper surface of the membrane was gently scraped to remove non-migrated cells and then washed with PBS. NIH3T3 cell line (ATCC, Manassas, VA, U.S.) served as a negative control for the chemotaxis assay. Migrated cells on the lower surface of the membrane were fixed with 4% PFA, stained with crystal violet, and manually counted in 5 randomly selected fields per well using an inverted microscope. Data are presented as the average number of migrated cells in 5 high-power fields (100×). Each experiment was performed in triplicate, and the data were averaged for statistical analysis.

### RNA extraction and real-time quantitative polymerase chain reaction

For detection of osteogenesis-related genes, rat mandibular bone marrow MSCs grown in osteogenic media were treated with 10^−7 ^mol/L NE with or without adrb3 siRNA. Cells maintained in osteogenic media served as controls. For detection of migration-related genes, rat mandibular bone marrow MSCs grown in basal media were treated with 10^−7 ^mol/L NE, with control siRNA or abrd3 siRNA. Cells maintained in basal media served as controls. Total RNA was extracted using an RNeasy Mini Kit (Qiagen, Alameda, CA, U.S.) in accordance with the manufacturer’s instructions. All the RNA samples were converted into cDNA for the same experiment to ensure that the efficiency of reverse transcription would be the same. cDNA synthesis was performed using a Revert Aid First Strand cDNA Synthesis Kit (Takara Bio Inc. Otsu, Japan). All the cDNA samples were analyzed in duplicate using a SYBR Premix Ex Taq Kit (Takara) on a quantitative PCR System (Applied Biosystems, Foster City, CA, U.S.). Primer sets were used to examine alkaline phosphatase (*ALP*), osteocalcin (*OCN*), and runt-related transcription factor-2 (*RUNX2*) for osteogenic differentiation and matrix metalloproteinase-2 (*MMP-2*) and tissue inhibitor of metalloproteinase-3 (*TIMP-3*) for migration. Glyceraldehyde 3-phosphate dehydrogenase (*GAPDH*) was served as an endogenous control. All primers were synthesized by Sangon Biotech (Shanghai, China). Amplification was performed under the following conditions: 40 cycles of 3 s at 95°C and 30 s annealing/extension at 60°C. Quantification of gene expression was based on the threshold cycle (CT) value for each sample calculated as the average of three replicate measurements using 7500 System SDS Software 1.3.1 (Applied Biosystems). The relative expression levels of each gene were normalized to those of *GAPDH*. The primer sequences are shown in Table 1.

**Table 1:** Real-time quantitative polymerase chain reaction primer sequences.

*Gene*	*Forward (5′-3′)*	*Reverse (5′-3′)*
Alkaline phosphatase (*ALP*)	AACGTGGCCAAGAACATCATCA	TGTCCATCTCCAGCCGTGTC
Osteocalcin (OCN)	GGTGCAGACCTAGCAGACACCA	AGGTAGCGCCGGAGTCTATTCA
Runt-related transcription factor-2 (*RUNX2*)	ACAGCCTCTTCAGCACAGTG	AACTCTTGCCTCGTCCACTC
Matrix metalloproteinase-2 (*MMP-2*)	CCCCTATCTACACCTACACCAAGAAC	CATTCCAGGAGTCTGCGATGAGC
Tissue inhibitor of metalloproteinase-3 (*TIMP-3*)	TCTGCAACTCCGACATCGTG	CGGATGCAGGCGTAGTGTT
Glyceraldehyde 3-phosphate dehydrogenase (*GAPDH*)	GGCACAGTCAAGGCTGAGAATG	ATGGTGGTGAAGACGCCAGTA

### Statistical analysis

Data are presented as the means ± SEM. Significance was assessed by independent two-tailed Student’s *t*-test for two-group comparison or one-way analysis of variance (ANOVA) followed by Dunnett’s test for multiple comparisons using SPSS 13.0. Probabilities of less than 0.05 were accepted as significant (*P<0.05).

## Results

### In vivo sympathetic innervation of bone marrow MSCs in rat mandibles

**Figure 2:**
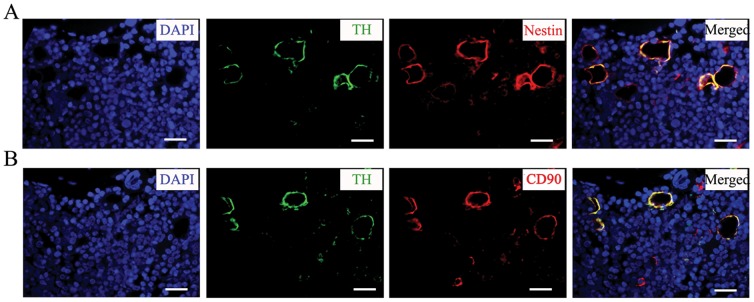
*In vivo* sympathetic innervation of bone marrow mesenchymal stem cells (MSCs) in rat mandibles. Cross sections were taken from the upper border of the anterior ramus to the inferior border of the rat mandible. Immunofluorescence staining shows a high degree of overlap of sympathetic nerve marker TH and MSC markers (**A**) nestin and (**B**) CD90 in continuous sections of normal rat mandibular bone marrow. Bar = 25 µm.

Because perivascular regions contribute to a critical stem cell niche for bone marrow MSCs, and because the sympathetic nervous system has been shown to thoroughly innervate the skeletal system along blood vessels [12]–[18], it was here determined whether bone marrow MSCs resided along sympathetic nerves in the normal rat mandible. Nestin and CD90 were used as MSC marker proteins to represent MSC population. Nestin^+^ cells (Figure 2A) and CD90^+^ cells (Figure 2B) were abundant in perivascular regions, and sympathetic fiber marker TH staining was intense along blood vessels (Figure 2A, B). Both nestin and CD90 were expressed in perivascular regions, and overlapped with TH expression closely (Figure 2A, B). These data indicate that nestin^+^ cells are indeed jaw bone marrow MSCs, and they are confined by sympathetic nerves under normal circumstances.

### In vivo effects of TCST on sympathetic mediator NE and its receptor abrd3

**Figure 3:**
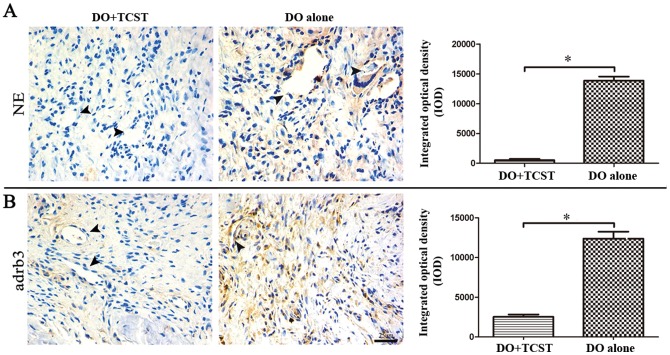
*In vivo* effects of TCST on sympathetic mediator norepinephrine (NE) and its receptor adrb3. NE and adrb3 expression in the callus after 10-d distraction was determined by immunohistochemistry. (**A**) NE expression was barely detectable in the distraction zone of the DO+TCST group, but it was still abundant in the DO-alone group. (**B**) Adrb3 expression was present in the DO+TCST group but to a much lower degree than in the DO-alone group. Arrowheads indicate perivascular regions. Scale bar = 25 µm. All experiments had been repeated 3 times, and equivalent final results were obtained each time. Data represent the mean ± SEM (n = 5). *P<0.05 by Students *t*-test.

Because NE is a major neurotransmitter of sympathetic nervous system, it is here hypothesized that sympathetic nerves may regulate bone marrow MSC mobilization *via* NE/abrd3. To test this, DO+TCST and DO-alone animal models were constructed. All rats recovered well from surgery and were available for immunohistochemical analysis. After 10 d of distraction, NE expression was barely detectable in the distraction zone of the rats in the DO+TCST group, but it was still abundant in the DO-alone group. IOD of the NE immunostaining in DO+TCST group was markedly lower than in the DO-alone group (Figure 3A). Similarly, adrb3 expression in the DO+TCST group was still present but to a much less pronounced degree than in the DO-alone group (Figure. 3B). MSCs were found to lack expression of other adrenergic receptor subtypes (data not shown). These data demonstrate that the TCST procedure depletes the expression of NE and adrb3 in DO callus.

### In vivo migration of MSCs from the perivascular area to the bone-forming area under distraction stress conditions

**Figure 4:**
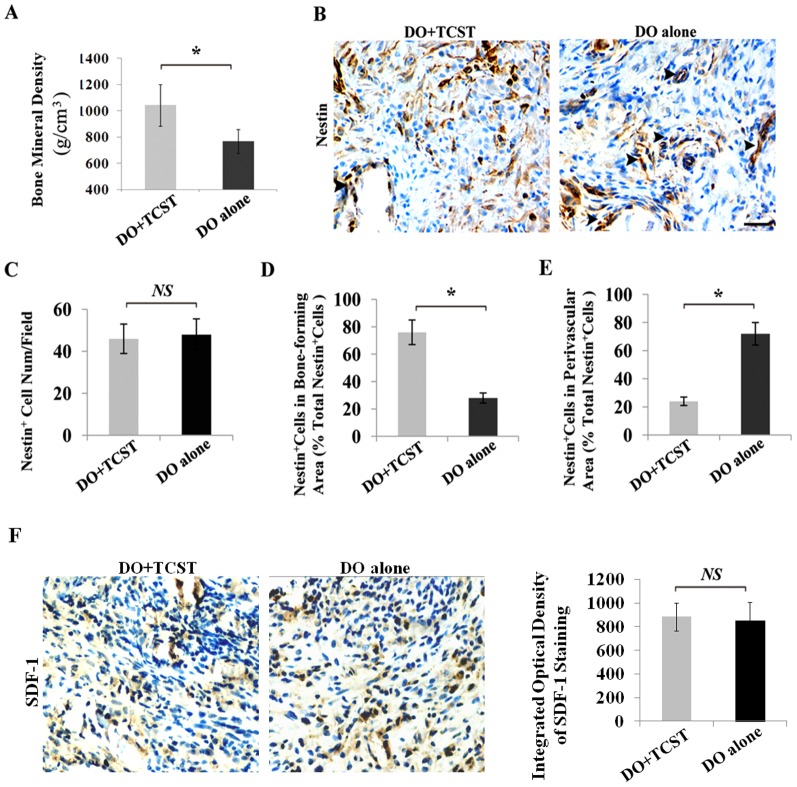
*In vivo* migration of MSCs from perivascular area to bone-forming area under distraction stress conditions. MSCs in the callus of each group were determined using nestin immunohistochemistry. (**A**) Sympathetic denervation increased the bone mineral density of mandibles. (**B**) Nestin^+^ cells were widely distributed in bone-forming area of the callus in DO+TCST group, but in the DO-alone group, nestin^+^ cells were mainly distributed in the vascular area. **(C)** Histograms showed no significant difference in the total number of nestin^+^ cells between the DO+TCST and DO-alone groups. (**D, E**) In nestin^+^ cells, the ratio of those in bone-forming area to those in the perivascular area, was markedly higher in the DO+TCST group than in the DO-alone group. (**F**) Immunohistochmistry showed that there to be no significant difference in the expression of SDF-1 between the DO+TCST and DO-alone groups. Arrowheads indicate the vascular area. Scale bar = 25 µm. Data represent the mean ± SEM (n = 5). *P<0.05 by Students *t*-test.

Sympathetic denervation improved bone mineral density in a rat model of mandibular DO (Figure. 4A), as in a previous study by the same team [26]. In order to further determine whether nestin^+^ MSCs have a greater tendency to migrate from the perivascular area under distraction stress after TCST than without TCST, nestin^+^ MSC distribution in the DO-gap was evaluated using immunohistochemistry. Nestin^+^ cells were widely distributed in bone-forming area of the callus in DO+TCST group, but the nestin^+^ cells in the DO-alone group were mainly distributed in the perivascular area rather than in the bone-forming area (Figure. 4B). There was no marked difference in the total number of nestin^+^ cells between the DO+TCST and DO-alone groups (Figure. 4C). The ratio of the number of nestin^+^ cells in bone-forming areas per total number of nestin^+^ cells was markedly higher in the DO+TCST group than in the DO-alone group (Figure. 4D). However, the ratio of nestin^+^ cells in the perivascular area per total number of nestin^+^ cells was markedly lower in the DO+TCST group than in the DO-alone group (Figure 4E). Immunohistochmistry showed there to be no significant difference in the expression of SDF-1, a well-documented chemotaxis factor for MSCs, between the DO+TCST and DO-alone groups (Figure 4F). These data indicate that MSCs have a higher tendency to migrate from the perivascular area to the bone-forming area under distraction stress with denervation of sympathetic nerves than without denervation.

### In vitro effects of NE on MSC osteogenic differentiation

**Figure 5:**
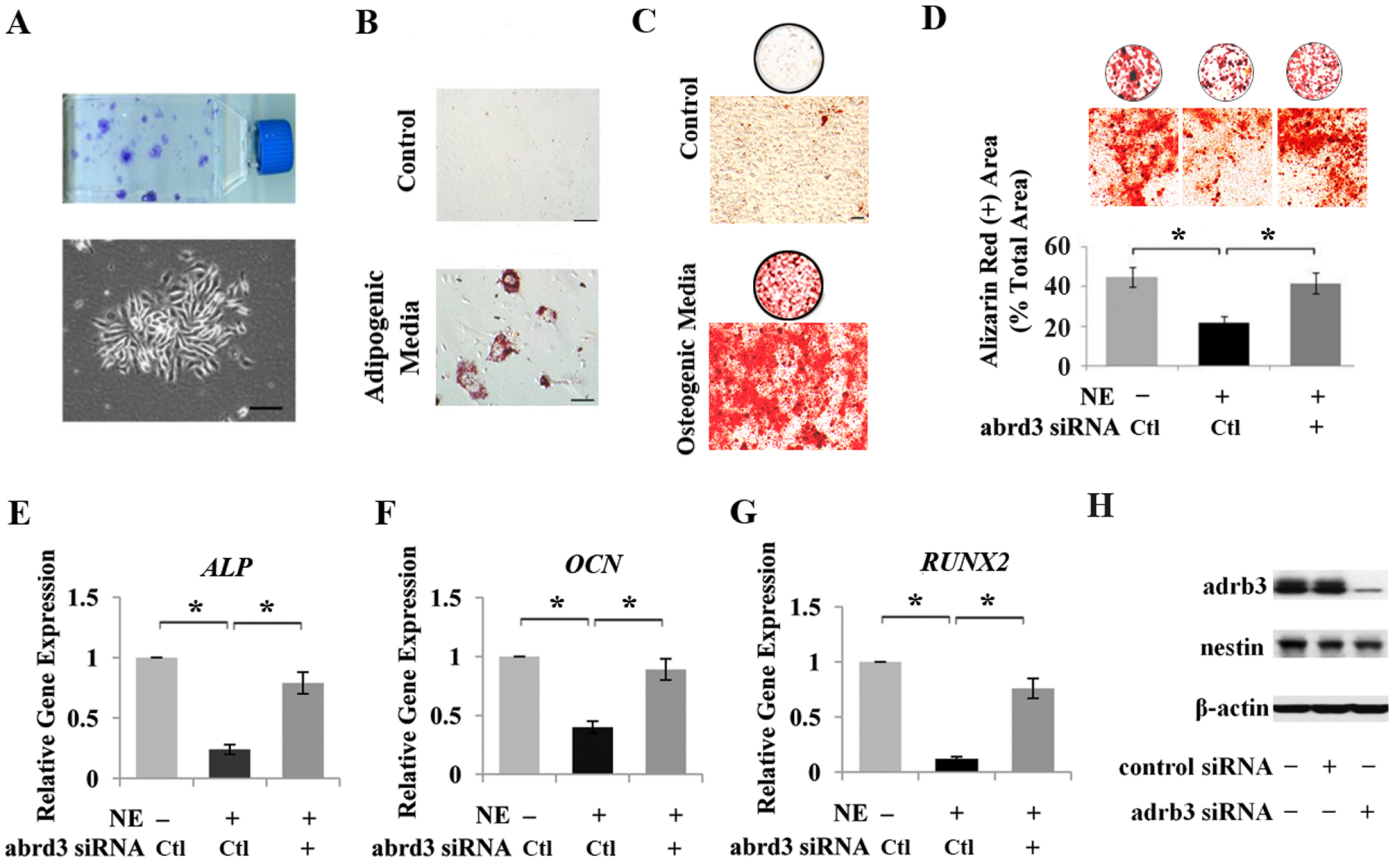
*In vitro* effects on NE to MSC osteogenic differentiation. (**A**) MSCs were isolated from rat mandible bone marrow, and formed colony-forming units-fibroblastic. (**B, C**) Mandibular MSCs could differentiate into osteoblasts and adipocytes, as indicated by Alizarin Red and Oil Red staining, respectively. (**D**) Alizarin Red staining showed that NE-treated MSCs had markedly less ability to form mineralized nodules than control MSCs, but knockdown of the *adrb3* gene using siRNA abolished the NE-induced inhibition of osteogenesis. (**E**–**G**) The expression of osteogenic marker genes *ALP*, *OCN* and *RUNX2* was measured using qPCR analyses in MSCs treated with NE relative to control cells and normalized to *GAPDH*. (**H**) Western blot analysis confirmed the specificity of adrb3 siRNA. Scale bar = 25 µm. Data represent the mean ± SEM of 5 independent experiments. *P<0.05 by ANOVA followed by Dunnett’s test.

In order to confirm whether NE affects MSC differentiation capabilities, MSCs were isolated from rat mandible bone marrow and cultured with expansion medium. MSC characterization showed that the jaw bone marrow MSCs could form colony-forming units-fibroblastic (CFU-F), differentiate into osteoblasts and adipocytes (Figure 5A–C), and express MSC markers, including CD90, CD73, CD44, and Sca-1 (data not shown). Under osteogenic culture conditions, NE-treated MSCs show a markedly decreased ability to form mineralized nodules than control MSCs, as indicated by Alizarin Red staining (Figure 5D). To further prove that adrb3 is indeed a functional mediator of NE signaling in MSC differentiation, the *adrb3* gene was knocked down in MSCs using siRNA, and it was found to abolish the NE-induced inhibition of osteogenic differentiation (Figure 5D). To further evaluate the differentiation cascade affected by the NE/adrb3 signaling, expression of several osteogenic differentiation genes, including *ALP*, *OCN*, and *RUNX2*, were investigated. They were found to be downregulated by NE, as determined by quantitative PCR (Figure 5E–G). Knockdown of the *adrb3* gene abolished the NE-induced inhibition of these osteogenic differentiation genes (Figure 5E–G). To confirm the efficacy and specificity of adrb3 siRNA, Western blot was used to show that adrb3 siRNA inhibited adrb3 expression in MSCs effectively and specifically (Figure 5H). In 3 experiments that were repeated, an average of 94% of the cells exhibited GFP expression as evaluated visually by fluorescence microscopy. These data suggest that NE can negatively regulate MSC osteogenic differentiation *via* adrb3 *in vitro*.

### In vitro effects of NE to MSC migration

**Figure 6:**
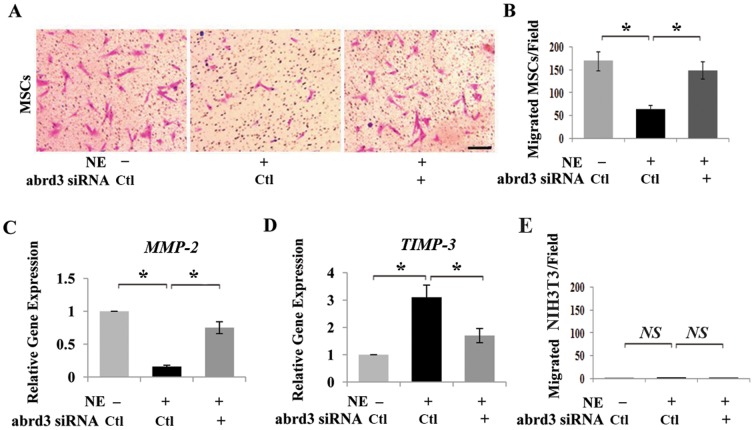
*In vitro* effects of NE to MSC migration. The migration of NE-treated MSCs towards stroma-derived factor-1 (SDF-1) was observed using a Transwell assay. Migrated cells were stained with crystal violet and counted. (**A, B**) NE treatment markedly decreased the number of MSCs that migrated to the undersurface of the membrane, and siRNA knockdown of the *adrb3* gene abolished the NE-induced inhibition of MSC migration. (**C, D**) Expression levels of the migration-related gene *MMP-2* and anti-migration gene *TIMP-3* were measured using qPCR analyses in MSCs treated with NE, as expressed relative to control and normalized to *GAPDH*. (**E**) The NIH3T3 cell line was used as a negative control for the chemotaxis assay. SDF-1 had no effect on NIH3T3 migration and this was not affected by NE. Scale bar = 25 µm. Data represent the mean ± SEM of three independent experiments. *P<0.05 by ANOVA followed by Dunnett’s test.

Next, a chemotaxis assay was used to determine the effects of NE on the migration capability of MSCs *in vitro*. A Transwell migration system was used to study the migration of MSCs in response to 25 ng/mL SDF-1. The number of migrated MSCs was markedly lower in the NE-treated group than in the control group, but knockdown of the *adrb3* gene using siRNA abolished the NE-induced inhibition of MSC migration (Figure 6A, B). To further evaluate the migration cascade affected by the NE/adrb3 signaling, the expression of migration-related gene *MMP-2* was found to be downregulated and the anti-migration gene *TIMP-3* was found to be upregulated by NE, as determined by quantitative PCR (Figure 6C, D). Knockdown of the *adrb3* gene abolished the NE-induced changes in the expression of these genes (Figure. 6C, D). The negative control for the chemotaxis assay, the NIH3T3 cell line showed no migration toward SDF-1, and NE showed no effects on NIH3T3 migration (Figure 6E).

In summary, blockage of NE by TCST can increase the mobilization of MSCs in rat mandibular DO. NE can inhibit migration and osteogenic differentiation capabilities of MSCs *via* adrb3 *in vitro*.

## Discussion

Nerve systems play an important role in bone metabolism and remodeling [26], [28]–[30]. Studies have demonstrated that the sympathetic nervous system is linked to leptin-responsive hypothalamic neurons, which could directly down-regulate bone remodeling *via* osteoblast β-ARs pathway *in vivo* [31]–[33]. However, it remains unclear whether NE regulates MSC mobilization in bone regeneration. Our previous study showed that sympathetic denervation could improve bone formation in rat mandibular DO [26]. However, the underlying mechanism remains unclear. In this follow-up study, we demonstrate that sympathetic denervation may induce MSC mobilization in rat mandibular DO, which is associated with inhibition of MSC migration and osteogenic differentiation by NE/adrb3 *in vitro*.

It has been demonstrated that mechanical stimulus increases the number and osteogenic capacity of osteoblasts that originate from MSCs from adjacent bone marrow and periosteum, and that administration of either ordinary MSCs or genetically modified MSCs can increase the amount of osteogenesis in DO [7]–[8], [34]–[37]. In this way, the abundance and mobilization of MSCs are crucial to bone formation in DO. Because sympathetic fibers and MSCs are closely related to the formation of blood vessels, it was here presumed that jawbone MSCs could be innervated by the sympathetic nervous system. To confirm this, the distribution of MSCs and sympathetic nerves was evaluated by immunofluorescence in rat mandibles using nestin and CD90 as MSC markers and TH as a sympathetic fiber marker. Results showed that MSCs in rat mandibles had considerable sympathetic innervation, indicating that bone marrow MSC niches could be controlled by sympathetic nerves. These results are consistent with previous studies on long bone sympathetic innervation [10].

Recent studies showed that the sympathetic nervous system could down-regulate SDF-1 expression in bone marrow to induce HSC mobilization from their osteoblast niches to peripheral circulation *via* β-ARs signaling pathways [21], [25]. Because the niches of MSCs reside in perivascular regions and sympathetic nerve fibers are associated with blood vessels [12]–[15], [18], [23], it is possible that sympathetic nerves can regulate MSCs mobilization by altering their perivascular niches. To confirm the involvement of sympathetic nerves in MSC mobilization in DO, a DO+TCST rat model was here established to show that the expression levels of NE and its receptor adrb3 were markedly decreased after surgery. These results suggested that sympathetic tone decreased considerably after TCST. In this study, nestin served as a marker of MSCs. Results showed that the relative number of perivascular nestin^+^ MSCs was markedly lower in the DO+TCST group than in the control group, suggesting that the normal level of sympathetic tone may maintain MSC quiescence in perivascular niches, and suppression of NE secretion may increase the rate of detachment of MSCs from their perivascular niches and the rate of migration to ossification zones under mechanical loading conditions.

NE has been shown to directly regulate cell activation, DNA synthesis, and differentiation [38]–[41]. To confirm the effects of NE on the properties of MSCs, the alternation of MSC osteogenic differentiation was investigated during co-culture with NE. These data suggest that NE suppresses osteogenic differentiation *in vitro*, and it may be caused by inhibition of traditional osteogenic genes such as *ALP*, *OCN*, and *RUNX2*. SDF-1 is a classic chemokine and known to be involved in MSC migration [42], [43]. Here, there was no significant difference in the expression of SDF-1 between the DO+TCST and DO-alone groups. However, when MSCs were either pretreated with NE or left untreated and placed in an SDF-1-dependent Transwell system, the results suggested that NE directly inhibits MSC migration *in vitro*, at least partially by downregulating *MMP-2* and upregulating *TIMP-3*. This has been shown to involve MSC migration [44]. Results indicate that NE keeps bone marrow MSCs from leaving their perivascular niches and from mobilizing. However, the details of the mechanisms by which NE inhibits MSC migration require further investigation. Other intracellular pathways that may be involved in this effect must be identified. It would also be interesting to further explore the role of NE in the immunomodulatory properties of MSCs [45], [46].

This is the first report to show that inhibition of MSC migration and osteogenic differentiation by neurotransmitter NE *in vitro* is associated with sympathetic denervation-induced MSC mobilization in rat mandibular DO. Modulation of the sympathetic outflow to the stem cell niche may be a novel means of enhancing bone formation. These findings may provide insight into the relationship between MSC mobilization and sympathetic nervous system in wide spectrum of bone regeneration processes.
